# Mitochondrial Damage in Sepsis

**DOI:** 10.14789/jmj.JMJ24-0016-P

**Published:** 2024-07-26

**Authors:** RICARD FERRER, TOSHIAKI IBA

**Affiliations:** 1 Intensive Care Department, Hospital Universitari Vall d’Hebron Universitat Autònoma de Barcelona, Barcelona, Spain; 1 Intensive Care Department, Hospital Universitari Vall d’Hebron Universitat Autònoma de Barcelona, Barcelona, Spain; 2 Department of Emergency and Disaster Medicine, Juntendo University Graduate School of Medicine, Tokyo, Japan; 2 Department of Emergency and Disaster Medicine, Juntendo University Graduate School of Medicine, Tokyo,Japan

**Keywords:** sepsis, mitochondria, programmed cell death, organ dysfunction, oxidative stress

## Abstract

Mitochondria not only generate adenosine triphosphate (ATP) and act as the powerhouse of the cell but also contribute to host defense by producing reactive oxygen species. Therefore, mitochondrial damage in sepsis directly results in a shortage of energy currency and dysregulation of the immune system. Other than those, mitochondrial damage results in the release of highly dangerous mitochondrial DNA, facilitating acidosis by modulating the metabolism and inducing programmed cell death, thereby facilitating disease progression in sepsis. Various forms of cell death are induced by mitochondrial damage. Aponecrosis is a secondary conversion from apoptosis to necrosis. Although apoptosis is initially intended, it cannot be completed due to ATP depletion from mitochondrial damage, ultimately leading to inflammatory necrosis. Besides such accidental cell death, programmed inflammation-inducing cell deaths such as necroptosis, ferroptosis, and pyroptosis are induced by mitochondrial damage in sepsis. Based on these findings, the regulation of mitochondrial damage holds promise for the development of new therapeutic approaches for sepsis.

## Introduction

Mitochondria are essential components of living cells that generate energy currency, control metabolic pathways, regulate intracellular calcium levels, produce reactive oxygen species, and modulate programmed cell death^1)^. Mitochondrial function is readily compromised in sepsis and significantly contributes to the worsening of the disease condition. In this perspective, we briefly overview recent advances in mitochondrial research related to sepsis.

## Impaired cellular respiration

**Figure 1:**
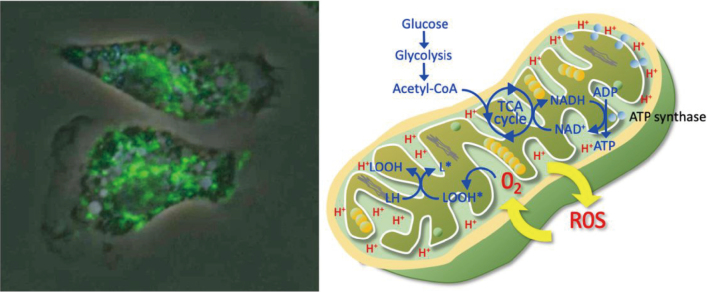
Immunofluorescent staining of mitochondria of the leukocytes and their functions Immunofluorescence of the leukocytes from rats displaying a mitochondrial fluorescein isothiocyanate (FITC) staining pattern of cellular distribution (left). Mitochondria are most densely distributed around the nucleus. The function of mitochondria is summarized in the right panel.

Mitochondria is responsible for producing adenosine triphosphate (ATP) via oxidative phosphorylation and functioning as the powerhouse of the cell (Figure 1). Damage to mitochondria impairs ATP production, leading to energy deficits in cells, particularly in high-energy-demanding organs such as the heart, kidneys, and liver. The count of mitochondria is considerably different between the cell types, and the mean volume for mitochondria from the rat was reportedly 0 60 μ^3^ in the heart, 0.42 μ^3^ in the liver, and 0.23 μ^3^ in the kidney cortex^2)^. Disrupted mitochondrial function is considered the fundamental mechanism of organ dysfunction, such as myocardial dysfunction and acute kidney injury in sepsis, and avoiding the decrease in the number and impaired function of mitochondria is crucial for maintaining cellular and organ functions^3, 4)^.

## Increase in oxidative stress

Reactive oxygen species (ROS) is a physiological byproduct of metabolism and is essential to the functions of immune systems. However, damaged mitochondria are known to produce excessive ROS, and overproduced ROS under pathophysiological conditions like sepsis are involved in the progression of the diseases. ROS can harm cellular components, including organelles, lipids, proteins, and DNA. Subsequent ROS-induced impaired cellular functions and inflammatory responses may result in organ damage. A complex system of interacting physiological antioxidant defenses normally diminishes oxidative stress and prevents damage to the mitochondria^5)^. However, since oxidative stress- mediated injury increases in sepsis, overwhelming the antioxidant defenses and facilitating the development of cellular damage and organ failure, antioxidant supplementation has been considered a potentially beneficial treatment for sepsis. The effects of multiple agents, including vitamins E and C, melatonin, N-acetylcysteine, selenium, carnosine, fish oil, and glutathione, have been examined^6)^. Nevertheless, the efficacy of these substances has not yet been established.

## Release of mitochondrial DNA

Mitochondrial damage can trigger the release of mitochondrial DNA (mtDNA) and other mitochondrial components into the cytoplasm and extracellular space, which act as a damage-associated molecular pattern (DAMP), inducing systemic toxicity and damage to multiple organs^7)^. Since mitochondria are suggested to originate from gram-negative bacteria and coexist in the cell (endosymbiotic theory), this endogenous ‘enemy’ acts as a pathogen-associated molecular pattern (PAMP) to induce hyper-proinflammatory reactions^8)^. It was reported that plasma levels of mtDNA are associated with the clinical outcome of septic shock and were suggested to be a better biomarker than lactate in predicting mortality in severe sepsis^9)^. Recently, mitochondrial dysfunction and the release of mtDNA have attracted attention due to their relevance to skeletal muscle weakness, known as ICU-acquired weakness^10)^.

## Metabolic dysfunction

Mitochondrial dysfunction disrupts cellular metabolism, leading to metabolic imbalances. First, glycolysis is intensified in carbohydrate metabolism; the failure to enter pyruvate into the tricarboxylic acid (TCA) cycle increases lactate generation. Second, mitochondrial dysfunction also affects lipid metabolism, and lipolysis is upregulated, increasing fatty acids and triglycerides. Impaired fatty acid oxidation and shifting towards anaerobic glycolysis result in further accumulation of lactate and metabolic acidosis. Third, changes in lipid metabolism also affect the levels of ketone bodies and amino acids^11)^. Other than the above, mitochondrial damage also affects calcium metabolism. Since mitochondria store a large amount of calcium, mitochondrial damage affects cellular signaling pathways, leading to increased cytosolic calcium levels. Elevated cytosolic calcium can activate various calcium-dependent enzymes, inducing cell death pathways and leading to reduced ATP production and further cellular stress^12)^. These metabolic changes are an important area of research for developing novel therapies for sepsis.

## Regulation of programmed cell death

Mitochondria can pursue programmed apoptosis, which can convert secondary to necrosis (aponecrosis), a form of uncontrolled cell death that further promotes inflammation and tissue damage. The mechanisms of apoptosis are explained as follows: first, in response to pro-apoptotic signals, mitochondria release cytochrome c from the intermembrane space into the cytosol. Cytochrome c binds to apoptotic protease activating factor-1 in the cytosol, leading to the formation of the apoptosome^13)^. This complex then recruits and activates procaspase-9, which initiates a cascade of downstream caspase activations, including caspase-3, -6, and -7. These executioner caspases dismantle the cell by cleaving various structural and regulatory proteins. Apoptosis is a silent form of cell death that does not impair organ function. However, since the apoptotic process requires sufficient ATP, a lack of ATP production by the mitochondrial damage causes apoptosis to transition into necrosis^14)^. In other words, apoptosis and necrosis partially share the same pathway, and apoptosis relies on adequate intracellular ATP levels. Meanwhile, when ATP is depleted, the cell death mechanism shifts to necrosis. Ultimately, mitochondrial damage in sepsis leads to an increase in necrotic cell death.

Mitochondrial damage can contribute to the initiation of necroptosis, a form of regulated inflammatory cell death that shares features with both apoptosis and necrosis. Increased production of ROS, the release of DAMPs, such as mtDNA and other mitochondrial components, and receptor-interacting protein kinases (RIPK) 1 and RIPK3 are involved in the induction of necroptosis^15)^. Necroptotic cells release DAMPs, triggering a robust inflammatory response similar to necrosis.

Mitochondrial damage also can contribute to the induction of ferroptosis, a form of regulated cell death distinct from apoptosis, necrosis, and necroptosis. Ferroptosis is characterized by iron-dependent lipid peroxidation. ROS produced by the damaged mitochondria promotes lipid peroxidation, a key feature of ferroptosis. Mitochondria play a crucial role in iron metabolism, including the storage and utilization of iron. Mitochondrial damage can disrupt iron homeostasis, leading to increased free iron within the cell. This free iron can catalyze the formation of highly reactive hydroxyl radicals via the Fenton reaction and induce ferroptosis^16)^.

Mitochondrial damage contributes to the induction of pyroptosis, which is characterized by the activation of inflammatory caspases. This leads to the cleavage of gasdermin D (GSDMD) and the subsequent formation of pores in the cell membrane. Increased ROS and DAMPs activate the NLRP3 (NACHT, LRR, and PYD domains-containing protein 3) inflammasome, which plays a critical role in the initiation of pyroptosis. mtDNA is recognized by NLRP3, leading to the activation of the inflammasome and subsequent pyroptosis^17)^.

## Organ dysfunction

The combined effects of energy deficits, increased oxidative stress, upregulated inflammation, and cell death induction contribute to the dysfunction of vital organs such as the heart, kidneys, lungs, and liver, a hallmark of severe sepsis and septic shock. Therefore, protecting and maintaining mitochondrial function is a promising strategy to improve organ damage in sepsis.

## Conclusion

In these two decades, the effects of impaired cellular function due to mitochondrial dysfunction on the development of organ dysfunction in sepsis have been unveiled. Impaired cellular respiration, increase in oxidative stress, release of mitochondrial DNA, metabolic dysfunction, and altered cell death mechanisms are the fundamental mechanisms of mitochondrial damage-induced cellular injury. Mitochondrial damage should be paid more attention to as a critical factor in the progression of sepsis. New research targeting the maintenance of mitochondrial function is warranted to pave the way for improved sepsis management.

## Funding

No funding was received.

## Author contributions

RF and TI wrote and reviewed the manuscript. Both authors read and approved the final manuscript.

## Conflicts of interest statement

The authors declare that they have no conflict of interest.

## References

[1] Li C, Wang W, Xie SS, et al: The Programmed Cell Death of Macrophages, Endothelial Cells, and Tubular Epithelial Cells in Sepsis-AKI. Front Med, 2021; 8: 796724. 10.3389/fmed.2021.796724 PMC8674574 34926535

[2] Gear AR, Bednarek JM: Direct counting and sizing of mitochondria in solution. J Cell Biol, 1972; 54: 325-345. 4339279 10.1083/jcb.54.2.325 PMC2108871

[3] Stanzani G, Duchen MR, Singer M: The role of mitochondria in sepsis-induced cardiomyopathy. Biochim Biophys Acta Mol Basis Dis, 2019; 1865: 759-773. 30342158 10.1016/j.bbadis.2018.10.011

[4] Sun J, Zhang J, Tian J, et al: Mitochondria in Sepsis-Induced AKI. J Am Soc Nephrol, 2019; 30: 1151-1161. 31076465 10.1681/ASN.2018111126 PMC6622414

[5] Nagar H, Piao S, Kim CS: Role of Mitochondrial Oxidative Stress in Sepsis. Acute Crit Care, 2018; 33: 65-72. 31723865 10.4266/acc.2018.00157 PMC6849061

[6] Sahoo DK, Wong D, Patani A, et al: Exploring the role of antioxidants in sepsis-associated oxidative stress: a comprehensive review. Front Cell Infect Microbiol, 2024; 14: 1348713. 38510969 10.3389/fcimb.2024.1348713 PMC10952105

[7] Supinski GS, Schroder EA, Callahan LA: Mitochondria and Critical Illness. Chest, 2020; 157: 310-322. 31494084 10.1016/j.chest.2019.08.2182 PMC7005375

[8] Kong C, Song W, Fu T: Systemic inflammatory response syndrome is triggered by mitochondrial damage (Review). Mol Med Rep, 2022; 25: 147. 35234261 10.3892/mmr.2022.12663 PMC8915392

[9] Kung CT, Hsiao SY, Tsai TC, et al: Plasma nuclear and mitochondrial DNA levels as predictors of outcome in severe sepsis patients in the emergency room. J Transl Med, 2012; 10: 130. 22720733 10.1186/1479-5876-10-130 PMC3441240

[10] Chatre L, Verdonk F, Rocheteau P, Crochemore C, Chrétien F, Ricchetti M: A novel paradigm links mitochondrial dysfunction with muscle stem cell impairment in sepsis. Biochim Biophys Acta Mol Basis Dis, 2017; 1863(10 Pt B): 2546-2553. 28456665 10.1016/j.bbadis.2017.04.019

[11] Wasyluk W, Zwolak A: Metabolic Alterations in Sepsis. J Clin Med, 2021; 10: 2412. 34072402 10.3390/jcm10112412 PMC8197843

[12] Harrington JS, Ryter SW, Plataki M, Price DR, Choi AMK: Mitochondria in health, disease, and aging. Physiol Rev, 2023; 103: 2349-2422. 37021870 10.1152/physrev.00058.2021 PMC10393386

[13] Power C, Fanning N, Redmond HP: Cellular apoptosis and organ injury in sepsis: a review. Shock, 2002; 18: 197-211. 12353919 10.1097/00024382-200209000-00001

[14] Eguchi Y, Shimizu S, Tsujimoto Y: Intracellular ATP levels determine cell death fate by apoptosis or necrosis. Cancer Res, 1997; 57: 1835-1840. 9157970

[15] Sureshbabu A, Patino E, Ma KC, et al: RIPK3 promotes sepsis-induced acute kidney injury via mitochondrial dysfunction. JCI Insight, 2018; 3: e98411. 29875323 10.1172/jci.insight.98411 PMC6124406

[16] Xl L, Gy Z, R G, N C: Ferroptosis in sepsis: The mechanism, the role and the therapeutic potential. Front Immunol, 2022; 13: 956361. 35990689 10.3389/fimmu.2022.956361 PMC9389368

[17] Dai S, Ye B, Zhong L, et al: GSDMD Mediates LPS-Induced Septic Myocardial Dysfunction by Regulating ROS-dependent NLRP3 Inflammasome Activation. Front Cell Dev Biol, 2021; 9: 779432. 34820388 10.3389/fcell.2021.779432 PMC8606561

